# Differences in electric field strength between clinical and non-clinical populations induced by prefrontal tDCS: A cross-diagnostic, individual MRI-based modeling study

**DOI:** 10.1016/j.nicl.2022.103011

**Published:** 2022-04-16

**Authors:** Yuki Mizutani-Tiebel, Shun Takahashi, Temmuz Karali, Eva Mezger, Lucia Bulubas, Irina Papazova, Esther Dechantsreiter, Sophia Stoecklein, Boris Papazov, Axel Thielscher, Frank Padberg, Daniel Keeser

**Affiliations:** a Department of Psychiatry and Psychotherapy, University Hospital LMU, Munich, Germany; b NeuroImaging Core Unit Munich (NICUM), Munich, Germany; c Department of Neuropsychiatry, Wakayama Medical University, Wakayama, Japan; d Clinical Research and Education Center, Asakayama General Hospital, Sakai, Japan; e Graduate School of Rehabilitation Science, Osaka Metropolitan University, Habikino, Japan; f Department of Psychiatry, Osaka University Graduate School of Medicine, Suita, Japan; g Department of Radiology, University Hospital LMU, Munich, Germany; h International Max Planck Research School for Translational Psychiatry (IMPRS-TP), Munich, Germany; i Department of Psychiatry and Psychotherapy, University of Augsburg, Germany; j Danish Research Centre for Magnetic Resonance, Centre for Functional and Diagnostic Imaging and Research, Copenhagen University Hospital Hvidovre, Copenhagen, Denmark; k Department of Health Technology, Technical University of Denmark, Kgs. Lyngby, Denmark; l Munich Center for Neurosciences (MCN) – Brain & Mind, 82152 Planegg-Martinsried, Germany

**Keywords:** Prefrontal tDCS, Structural MRI, Electric field, Major depressive disorder, Schizophrenia, Dorsolateral prefrontal cortex

## Abstract

**Introduction:**

Prefrontal cortex (PFC) regions are promising targets for therapeutic applications of non-invasive brain stimulation, e.g. transcranial direct current stimulation (tDCS), which has been proposed as a novel intervention for major depressive disorder (MDD) and negative symptoms of schizophrenia (SCZ). However, the effects of tDCS vary inter-individually, and dose–response relationships have not been established. Stimulation parameters are often tested in healthy subjects and transferred to clinical populations. The current study investigates the variability of individual MRI-based electric fields (*e-fields*) of standard bifrontal tDCS across individual subjects and diagnoses.

**Method:**

The study included 74 subjects, i.e. 25 patients with MDD, 24 patients with SCZ, and 25 healthy controls (HC). Individual e-fields of a common tDCS protocol (i.e. 2 mA stimulation intensity, bifrontal anode-F3/cathode-F4 montage) were modeled by two investigators using SimNIBS (2.0.1) based on structural MRI scans.

**Result:**

On a whole-brain level, the average e-field strength was significantly reduced in MDD and SCZ compared to HC, but MDD and SCZ did not differ significantly. Regions of interest (ROI) analysis for PFC subregions showed reduced e-fields in Sallet areas 8B and 9 for MDD and SCZ compared to HC, whereas there was again no difference between MDD and SCZ. Within groups, we generally observed high inter-individual variability of e-field intensities at a higher percentile of voxels.

**Conclusion:**

MRI-based e-field modeling revealed significant differences in e-field strengths between clinical and non-clinical populations in addition to a general inter-individual variability. These findings support the notion that dose–response relationships for tDCS cannot be simply transferred from healthy to clinical cohorts and need to be individually established for clinical groups. In this respect, MRI-based e-field modeling may serve as a proxy for individualized dosing.

## Highlights

• MDD and SCZ showed lower prefrontal tDCS-induced e-field strengths compared to HC.
• Average e-field strengths did not significantly differ between MDD and SCZ patients.
• Inter-individual variability of e-field intensities and distribution was prominent.
• Inter-rater variability emphasizes the importance of standardized positioning.

## 1 Introduction

Transcranial direct current stimulation (tDCS) is a transcranial electrical stimulation (tES) and non-invasive brain stimulation (NIBS) technique, used as experimental and therapeutic interventions to modulate cortical activity. tDCS of the prefrontal cortex (PFC) showed initial evidence of efficacy in psychiatric disorders, e.g. in major depressive disorder (MDD) (Brunoni et al., 2016, Moffa et al., 2020) and negative symptoms of schizophrenia (SCZ) (Valiengo et al., 2020, Yu et al., 2020). Compared to other NIBS methods, such as repetitive transcranial magnetic stimulation (rTMS), tDCS is less expensive, portable, and potentially suitable for all treatment settings, including home treatment (Palm et al., 2018). In contrast to rTMS, however, standard tDCS protocols provide a non-focal, less targeted stimulation and no individual adjustment of stimulation intensity (Bikson et al., 2016). Current tDCS protocols usually apply fixed intensities (e.g. 1 or 2 mA) and standardized electrode montages (e.g. defined by the international 10–20 EEG system).

However, it is questionable whether such standardized protocols are optimal for tDCS. Inter-individual variability of tDCS effects in motor and non-motor regions has generally been reported with standardized “one size fits all” applications (Wiethoff et al., 2014, Workman et al., 2020). Furthermore, therapeutic applications of tDCS with psychiatric patients showed considerable inhomogeneity in the treatment response (Lefaucheur et al., 2020). Wörsching et al. (2017) observed that active tDCS induced additional variability in resting-state connectivity compared with sham tDCS. Recent studies show several factors that affect the behavioral outcome of tDCS, such as the baseline resting-state functional connectivity (FC) (Cerreta et al., 2020) and concentration of the neurochemicals (Filmer et al., 2019). However, the true picture of these inter-individual response variations is not fully understood yet.

To account for the inter-individual variability in response to tDCS interventions, personalization is suggested in terms of intensities and targets. tDCS-induced electric field (e-field) has been proposed as a proxy for individual adjustment of tDCS intensity as it reflects the received dosage of the stimulation. Recent intracranial field measurements (Huang et al., 2017, Opitz et al., 2016) and modeling studies (Antonenko et al., 2021a, Laakso et al., 2015) demonstrated variability in e-field intensity across subjects. Inter-individual variation in e-field strength has been partially explained by variable structural (Mosayebi-Samani et al., 2021) and functional neuroanatomy (López-Alonso et al., 2014, Wiethoff et al., 2014) but is not yet completely understood. Recent machine learning study proposed precision dosing of tDCS derived from individual e-field characteristics, which predicted the responders of cognitive training (working memory improvement) with 86% accuracy (Albizu et al., 2020). To individualize the tES intensity, reverse-calculation e-field modeling recently showed a promising result (Caulfield et al., 2020).

The variation in electrode positioning also contributes to tDCS-induced e-field variability. Opitz et al. (2018) investigated the e-field distribution with surgical epilepsy patients and recommended keeping the electrode positioning error under 1 cm to achieve the desired e-field distribution. Five percent of electrode mislocalization at F3/F4 and M1/SO (1–1.5 cm drift with average head size) lead to a significant difference in e-field distribution. A validation of motor cortex localization based on C3/C4 locations with international 10–20 EEG demonstrated a low to fair intraclass correlation coefficient (ICC) between two independent raters. These e-field intensity variations due to a less precise electrode localization may be a source of variability in tDCS response.

For computational modeling of e-fields, SimNIBS (https://www.simnibs.de) is an established approach based on Finite-Element Method (FEM) (Thielscher et al., 2015). This free software package allows researchers to simulate tDCS application on subjects’ anatomical magnetic resonance imaging (MRI) scans. tDCS-induced e-fields are calculated by separating the different tissue types. SimNIBS stimulation simulation allows numerical statistical comparison of e-field strength, and it can visualize the e-fields distribution in the brain.

The present study investigates the variation of e-field strength and distribution for a standard protocol of prefrontal tDCS in MDD and SCZ, i.e. bifrontal anode-F3/cathode-F4 montage with 2 mA stimulation intensity (Bajbouj et al., 2018, Blumberger et al., 2012, Brunoni et al., 2013, Padberg et al., 2017) as left dorsolateral prefrontal cortex (DLPFC) plays an important role in the pathophysiology of MDD (Koenigs and Grafman, 2009) as well as negative symptoms of SCZ (Potkin et al., 2009). In order to imitate clinical practice, two blinded investigators placed the electrodes over F3 and F4 by calculating both positions based on nasion, inion, and mastoids coordinates. Thus, this study aims to characterize the cross-diagnostic and inter-individual variability of tDCS-induced e-fields and to test the assumption that dosage parameters can be readily transferred from non-clinical to clinical populations.

## 2 Method

### 2.1 Participants

All patients and HC were recruited in the Department of Psychiatry and Psychotherapy, University Hospital, LMU Munich, Germany. Data from 74 right-handed subjects were analyzed in the study divided into three groups: MDD (n = 25, male = 10, age: 38.1 ± 10.2 yrs, range: 22–56 yrs), SCZ (n = 24, male = 11, age: 36.9 ± 13.4 yrs, range: 20–59 yrs), and HC (n = 25, male = 13, age: 35.5 ± 11.1 yrs, range: 20–57 yrs). All MDD subjects had a primary DSM-5 diagnosis of Major Depressive Disorder and HDRS-21 (Hamilton Depression Rating Scale) score of ≥ 15. SCZ subjects were diagnosed with ICD-10 F20. None of the subjects reported a history of neurological disorder and none of the HC group had a psychiatric disease. Three subject groups were matched for age and gender. The study was approved by the local ethics committee. The study was conducted in accordance with the code of ethics of the world medical association (declaration of Helsinki). All participants gave their written informed consent.

### 2.2 MRI data acquisition

All subjects underwent T1-weighted structural MRI using a 3-Tesla MR-scanner equipped with a 20-channel head coil (Magneton Skyra, Siemens Healthineers, Erlangen, Germany). The participants wore earplugs for noise protection. T1-weighted images were acquired with a 3D magnetization-prepared fast gradient echo (MPRAGE) sequence (TR: 1900 ms, TE: 2.2 ms, flip angle: 9°, 0.8 mm^3^ isotropic voxels).

### 2.3 Electric field calculation

For MRI-based e-field modeling, we used SimNIBS (version 2.0.1; http://Simnibs.de/) (Thielscher et al., 2015); a free software that allows the calculation and simulation of the e-fields induced by tDCS or other NIBS. We applied SimNIBS in Ubuntu 16.04. environment. The respective software was required for the following SimNIBS procedure: FreeSurfer (version 6.0.0; https://surfer.nmr.mgh.harvard.edu/) (Dale et al., 1999, Fischl et al., 1999) and FMRIB Software Library (FSL) (version 6.0.0; https://fsl.fmrib.ox.ac.uk/fsl/fslwiki/) (Jenkinson et al., 2012, Smith et al., 2004, Woolrich et al., 2009). Additionally, we used some open source tools such as MeshFix (Attene, 2010, Attene and Falcidieno, 2006) for meshing and Get DP (Dular et al., 1998) for FEM computation.

Before SimNIBS was started, “mri2mesh” was used to generate an individual tetrahedral volume mesh of the head (Windhoff et al., 2013). To model prefrontal tDCS, a standard bipolar montage was used; anodal-F3/cathodal-F4 montage according to the international 10–20 EEG system. The electrode size was set to a rectangular with dimensions of 4.5 cm × 6.5 cm. We simulated the thickness of the electrodes as 5 mm and the saline-soaked sponges with a thickness of 6 mm. The current intensity was set to 2 mA and −2mA on the left and right hemispheres respectively. Conductivity was set as default settings of SimNIBS (WM: 0.126 S/m, GM: 0.275 S/m, CSF: 1.654 S/m, Skull: 0.010 S/m and skin: 0.465 S/m).

The localization of the electrodes was performed to imitate the clinical practice where F3/F4 locations are determined by measuring the head size using the locations of inion, nasion, and mastoids. For modeling, a python script was used which was developed by the SimNIBS developers. This script automatically calculated F3 and F4 coordinates by inserting individual inion, nasion, and mastoids coordinates. The direction of the electrodes was manually adjusted so that the sponges are in parallel to each other.

SimNIBS calculation was conducted independently by two blinded investigators (i.e. investigators 1 and 2). The outcome of the individual e-field distribution map was visualized using gmsh (Geuzaine and Remacle, 2009, Schöberl, 1997). SimNIBS software calculates the peak values of the e-field intensity (E) as a ratio of voltage divided by distance (E = V/m) at the 50th, 75th, 90th, 95th, 99th, and 99.5th percentiles of the voxels. For example, when the e-field value is indicated at the 90th percentile, it means that 90 percent of the voxels have an e-field intensity lower than its shown value (Opitz et al., 2015).

### 2.4 Transformation to volumetric space

The individual electric fields calculated with the SimNIBS were converted to volumetric space using the script “msh2nifti” developed by Nicholas Cullen (University of Pennsylvania, Neuroscience graduate group 2018, https://github.com/ncullen93/mesh2nifti/blob/master/msh2nifti.py). We have made some minor changes, such as integrating an input and output folder structure to the script, which otherwise did not change the script's content. msh2nifti was used to transform the grey matter (GM) to volumetric space. The voxel size was set to 2 mm.

### 2.5 Analyses and visualization

#### 2.5.1 Numerical statistical calculations

Numerical statistical calculations were performed using IBM SPSS Statistics (version 20.0.0.1, IBM Corp. Armonk, NY) and R Studio (version 1.2.5033, https://www.r-project.org/) (R Core Team, 2013). The Shapiro-Wilk test of normality showed that our e-field dataset is not normally distributed, therefore we used non-parametric statistical tests. Inter-rater reliability was tested using the ICC test. Kruskal-Wallis test and post-hoc Mann-Whitney test were applied to see the variance of electric field strength in SCZ, MDD, and HC. Age and gender were included as covariates. The significance level was Bonferroni corrected and set at 0.008 (0.05 divided by 6).

#### 2.5.2 Voxel-based whole-brain analysis

Voxel-wise whole-brain analysis was conducted using FSL randomize v2.9. Age and gender were inserted as covariates. Family-wise error (FWE) rate was controlled and only FWE-corrected p values of <0.05 were accepted as significant results. To assign and extract the voxels with significant results and anatomical regions where the voxels were located, we used the command “autoaq” in FSL. Clusters with more than 30 voxels are reported. For the atlas, we used the Talairach Daemon Labels (Lancaster et al., 1997, Lancaster et al., 2000, Talairach, 1988). Results on volumetric space were further registered to surface space using workbench v1.3.2 with the command ‘wb_command -volume-to-surface-mapping’ (https://www.humanconnectome.org/software/workbench-command/-volume-to-surface-mapping) and projected onto the Conte69 surface template (Van Essen et al., 2011).

#### 2.5.3 Voxel-based ROI analysis in PFC

Using the Sallet atlas (Sallet et al., 2013), we placed our regions of interest (ROI) in 6 regions: Brodmann’s area (BA) 8B, 9, 9/46D, 9/46 V, 10 and 46. This selection was based on our secondary analysis of the Escitalopram versus Electrical Direct-Current Theror Depression Study (ELECT-TDCS; Brunoni et al. 2017) which showed an association of GM volume in PFC subregions and improvement of depression scores only after tDCS, but not after escitalopram or placebo (Bulubas et al., 2019). First, “fslstats” was used to extract non-zero voxels in the ROIs with binary masks. Based on these data, the maximum e-field within the ROIs was calculated and averaged across individuals in each ROI. The 50th and 75th percentile values of the averaged maximum e-fields were then used as the low-cut threshold. The number of voxels exceeding the threshold was calculated in each of the 6 ROIs for each threshold and investigator. Group differences between MDD, SCZ, and HC were calculated with the Kruskal-Wallis test and post hoc pairwise Wilcoxon test. The significance level was Bonferroni corrected.

## 3 Results

**Fig. 1:**
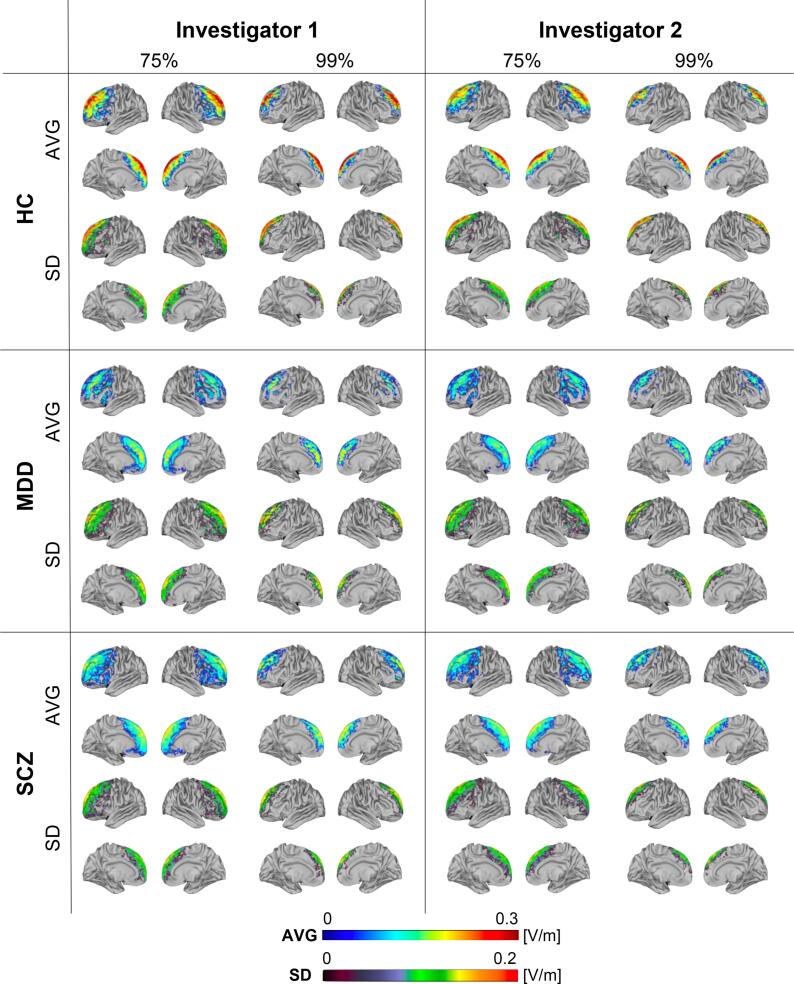
Distribution of average (AVG) e-field strength and standard deviation (SD). AVG and SD of the e-field distribution are illustrated for three groups (HC: healthy control, MDD: major depressive disorder, SCZ: schizophrenia), two intensity thresholds (75th and 99th percentile of the voxels) and two investigators (1 and 2).

The demographic characteristics of all subjects are shown in Table 1. No significant difference was observed among subject groups regarding the age, gender, and intracranial volume (ICV) (age; Kruskal Wallis test; Chi-square = 0.793, p = 0.673, df = 2 / gender; one-way ANOVA; F(2,71) = 0.976, p = 0.382 / ICV; chi-square test; X^2^(2, N = 74) = 0.72, p = 0.696). Fig. 1 illustrates the distribution of the e-field for the 75th and 99th percentile thresholds of the voxels for each subject group and rater. The e-field maximum was in PFC regions.

**Table 1:** Demographic and clinical characteristics of the study sample.

	**HC**	**MDD**	**SCZ**
	(n = 25)	(n = 25)	(n = 24)
**Age (range)**	20–57	22–56	20–59
**Age (mean ± SD)**	35.5 ± 11.28	38.1 ± 10.46	36.9 ± 13.71
**Male (%)**	13 (52%)	10 (40%)	11 (46%)
**ICV (cm^3^ ± SD)**	1558 ± 168	1558 ± 196	1623 ± 187
**BDI**	–	23.5 ± 10.27	–
**MADRS**	–	21.7 ± 6.97	–
**PANSS (total)**	–	–	54.8 ± 17.1

Abbreviations: HC = Healthy control, MDD = Major depressive disorder, SCZ = Schizophrenia, SD = Standard deviation, ICV = Intracranial volume, BDI = Beck’s Depression Inventory, MADRS = Montgomery-Asberg Depression Rating Scale, PANSS = Positive and Negative Syndrome Scale.

### 3.1 Numeric comparison of e-field strength between experimental groups and investigators

**Fig. 2:**
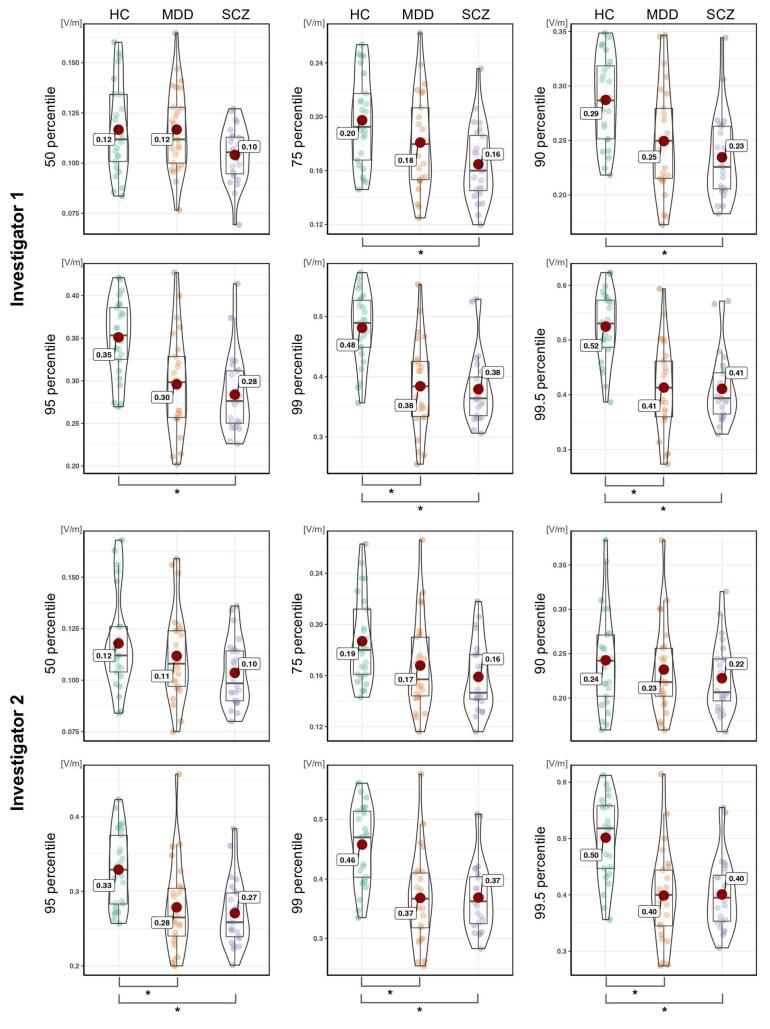
Group comparison of e-field intensity for both investigators, The vertical axis shows the e-field strength (V/m). The horizontal axis is the three subject groups (HC = healthy controls, MDD = major depressive disorder, SCZ = schizophrenia) separated for six percentile thresholds of the voxels (50%, 75%, 90%, 95%, 99% and 99.5%). The dots in each graph indicate every individual’s e-field value. * = p < 0.008 (0.05/6 - corrected for multiple comparison).

Using Kruskal Wallis tests, we observed a significant difference in electric field strength between MD, SCZ, and HC groups, which varied between both investigators. Investigator 1 observed a significant difference from the 75th percentile of the voxels and above, whereas investigator 2 observed a significant difference only above the 95th percentile. Significant differences were found between MDD and HC as well as SCZ and HC (post-hoc Mann-Whitney tests). However, the difference between MDD and SCZ did not reach significance at any percentile threshold (Fig. 2, Table 2).

**Table 2:** Statistical results of the e-field strength subject group comparison.

**Kruskal Wallis test**												
	50%		75%		90%		95%		99%		99.5%	
	X^2^	P	X^2^	P	X^2^	P	X^2^	P	X^2^	P	X^2^	P

Investigator 1	–	–	10.53	< 0.005	16.06	< 0.001	20.57	< 0.001	26.44	< 0.001	27.77	< 0.001
Investigator 2	–	–	–	–	–	–	16.6	< 0.001	21.85	< 0.001	22.45	< 0.001

**Post-hoc Mann–Whitney test**												

**MDD vs HC**												

	50%		75%		90%		95%	99%		99.5%		

U	P	U	P	U	P	U	P	U	P	U	P

Investigator 1	–	–	–	–	170	< 0.006	136	< 0.001	96	< 0.001	89	< 0.001
Investigator 2	–	–	–	–	–	–	145.5	< 0.001	111.5	< 0.001	108	< 0.001

**SCZ vs HC**												

	50%		75%		90%		95%	99%		99.5%		

U	P	U	P	U	P	U	P	U	P	U	P

Investigator 1	–	–	136	< 0.001	105	< 0.001	88	< 0.001	68	< 0.001	63	< 0.001
Investigator 2	–	–	–	–	–	–	112.5	< 0.001	92	< 0.001	90	< 0.001

Abbreviations: HC = Healthy control, MDD = Major depressive disorder, SCZ = Schizophrenia.

### 3.2 Global voxel-wise spatial comparison in whole brain (Group and inter-rater comparison)

**Fig. 3:**
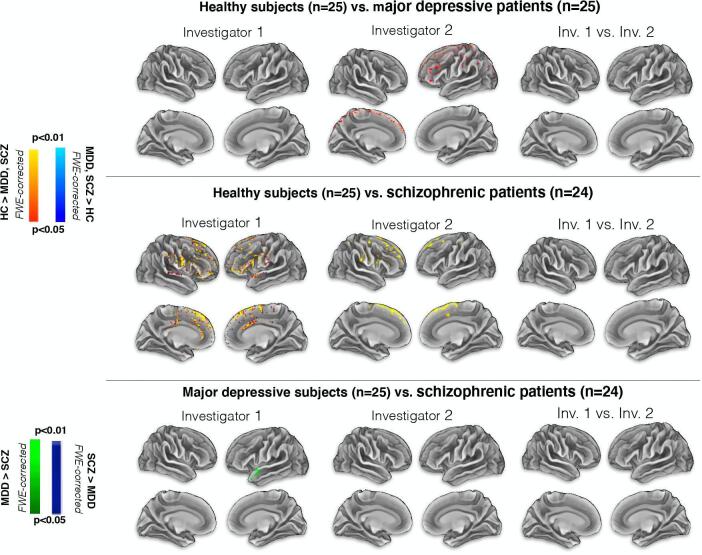
Comparison of the electric field intensity using volumetric data projected onto the surface space. For investigator 1 there was a significant difference between HC and SCZ as well as MDD and SCZ. For investigator 2 there was a significant difference between HC and MDD as well as HC and SCZ. Though these results differ between investigators, we observed no statistically significant difference between investigators 1 and 2.

Fig. 3 shows the comparison of e-field intensities between the groups as well as between investigators. Table 3 gives an overview of all brain regions included in the clusters with more than 30 voxels (FWE-corrected p < 0.05). We observed no significant differences between investigators, although subject group analysis revealed discrepant findings. Consistent findings by both investigators were the differences in e-field intensity between SCZ and HC located in frontal lobe regions; SCZ showed a weaker e-field bilaterally for the superior frontal gyrus and in the right middle frontal gyrus. Other discrepant findings for both investigators are shown in Table 3.

**Table 3:** Brain regions consisting the clusters.

**Investigator 1**										
						Main area in the cluster				
Direction of effect	Cluster	Number of voxels	X	Y	Z	Hemisphere	Lobe	Cortical area	GM/WM	Brodmann area
**HC > SCZ**	Cluster 1	20,210	−4	34	44	Right	Frontal	Superior Frontal Gyrus	WM	–
						Right	Frontal	Middle Frontal Gyrus	WM	–
						Left	Frontal	Superior Frontal Gyrus	WM	–
						Left	Frontal	Inferior Frontal Gyrus	WM	–
						Left	Frontal	Middle Frontal Gyrus	WM	–
**MDD > SCZ**	Cluster 1	283	−50	8	−20	Left	Temporal	Superior Temporal Gyrus	GM	38
						Left	Temporal	Superior Temporal Gyrus	GM	22
						Left	Temporal	Superior Temporal Gyrus	WM	–
	Cluster 2	81	−34	18	−30	Left	Temporal	Superior Temporal Gyrus	GM	38
						Left	Frontal	Inferior Frontal Gyrus	WM	–

**Investigator 2**										

						Main area in the cluster				
Direction of effect	Cluster	Number of voxels	X	Y	Z	Hemisphere	Lobe	Cortical area	GM/WM	Brodmann area

**HC > MDD**	Cluster 1	16,602	1	−2	26	Right	Frontal	Superior Frontal Gyrus	WM	–
						Right	Frontal	Middle Frontal Gyrus	WM	–
						Left	Frontal	Superior Frontal Gyrus	WM	–
						Left	Frontal	Middle Frontal Gyrus	WM	–
	Cluster 2	46	−36	−8	16	Left	Sub lobar	Insula	GM	13
						Left	Sub lobar	Insula	WM	–
	Cluster 3	43	–32	22	8	Left	Frontal	Sub Gyral	WM	–
						Left	Sub lobar	Insula	GM	13
						Left	Sub lobar	Extra Nuclear	WM	–
**HC > SCZ**	Cluster 1	4728	−4	26	58	Right	Frontal	Superior Frontal Gyrus	WM	–
						Right	Frontal	Middle Frontal Gyrus	WM	–
						Right	Frontal	Precentral Gyrus	WM	–
						Right	Frontal	Sub Gyral	WM	–
						Left	Frontal	Superior Frontal Gyrus	WM	–
	Cluster 2	80	32	−28	60	Right	Frontal	Precentral Gyrus	GM	4
						Right	Frontal	Precentral Gyrus	WM	–
						Right	Parietal	Postcentral Gyrus	GM	3
	Cluster 3	51	36	−10	8	Right	Sub lobar	Insula	WM	–
						Right	Sub lobar	Extra Nuclear	WM	–

Abbreviations: SCZ = Schizophrenia, MDD = Major depressive disorder, HC = Healthy control, GM = Grey matter, WM = White matter.

### 3.3 Local voxel-wise comparison in PFC regions

**Fig. 4:**
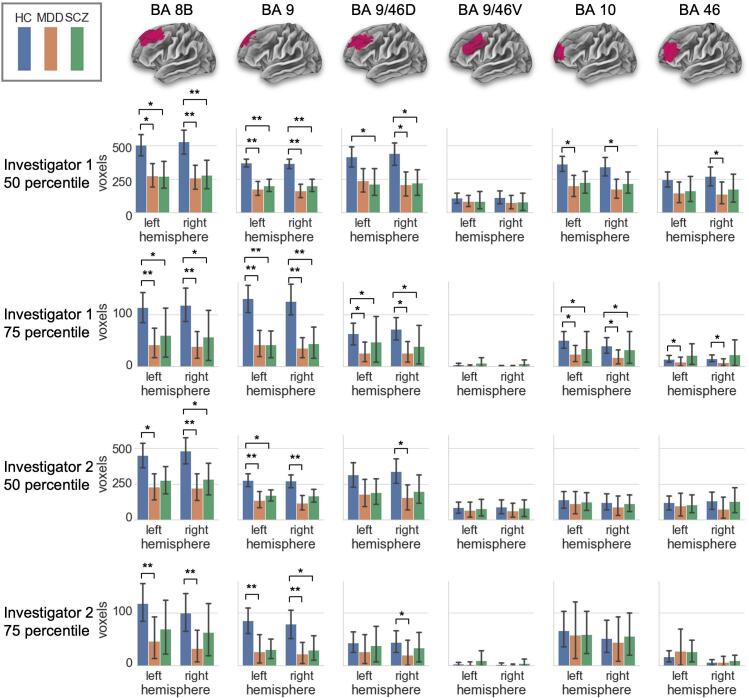
Group variability of e-field strength in PFC parcellated by Sallet atlas. The maximum e-field values in PFC were averaged among all subjects, and its 50th and 75th percentile values were used as the threshold. The graph shows the number of voxels which exceeded the threshold in each PFC area from both investigators 1 and 2. * = p < 0.007, ** = p < 0.0001.

Fig. 4 depicts the group variability of e-fields across PFC regions according to Sallet parcellation (Sallet et al., 2013). The graph shows the number of voxels at each PFC region that had an e-field value higher than the 50th or 75th percentile thresholds of the averaged total PFC e-field value across all subjects. A significant difference between groups was consistently observed by both investigators for BA 8B, 9, and 9/46D. A difference between MDD and HC was detected for bilateral BA 8B, 9, and right BA 9/46D regions, at both 50th and 75th thresholds. The difference between SCZ and HC was observed for right BA 8B and left BA 9 regions, at the 50th percentile threshold. However, the effect was only found for the right BA 9 at the 75th percentile threshold.

### 3.4 Inter-investigator difference

**Fig. 5:**
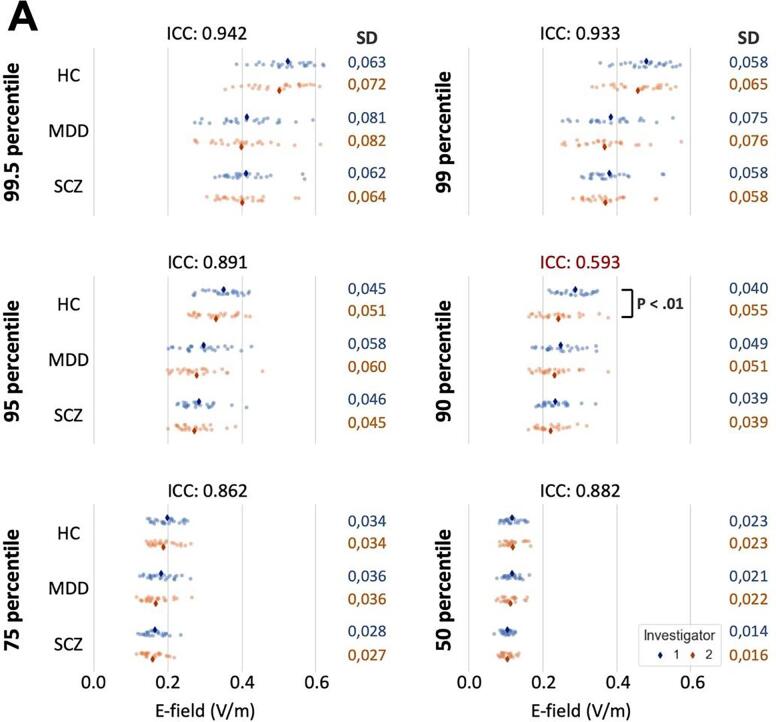

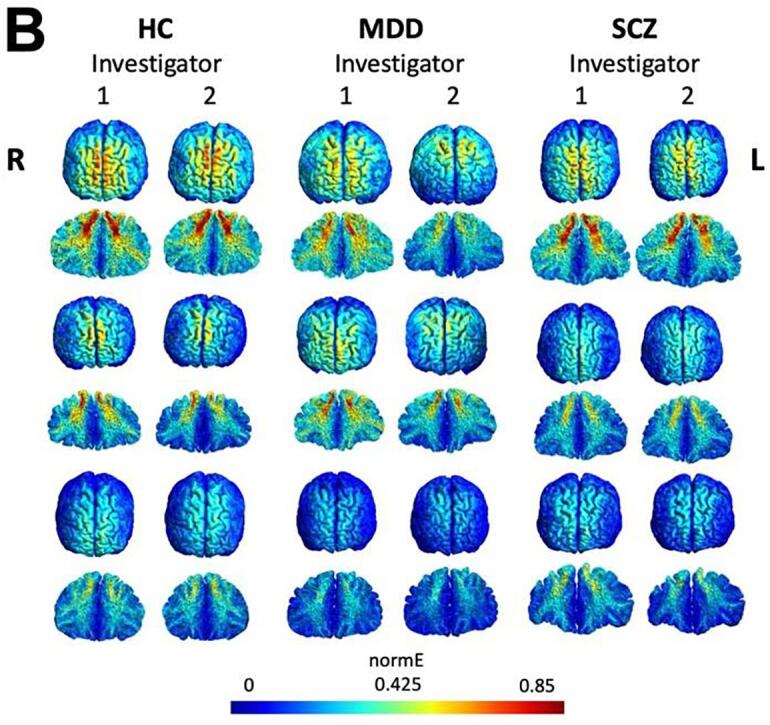
A) distribution of e-field value classified by subject groups, investigators, and percentile thresholds of the voxels. Every dot indicates an individual’s e-field value which falls at each percentile when all voxels are listed in the order of e-field strength. Graph with higher percentile shows higher standard deviation (SD) which indicates inter-individual differences of simulated e-field values. Intraclass correlation (ICC) was high except for 90th percentile values. B) Spatial e-field distribution from selected 3 subjects in each subject group simulated by 2 investigators. E-field strength is reflected in each subject’s individual space (whole-brain and sagittal view cut at the temporal pole). It shows that the e-field value of patients can be as high as HC, and HC may also have as low e-field as patients. Though the group difference is significant, the inter-individual difference is prominent.

Intraclass correlation between both investigators was strong, except for the 90th percentile of the voxels, where a significant difference was observed between investigators in HC data at the 90th percentile (p < 0.01) (Fig. 5A). The calculation of the euclidean distance showed a significant difference for the electrode positions XYZ applied by the two investigators. This was evident with the F3 electrode for HC (p < 0.01) and SCZ (p < 0.05) for the XYZ coordinates, and MDD (p < 0.05) for Y and Z coordinates (Table S1). With the F4 electrode, there was a significant Euclidean difference for Y and Z coordinates with MDD (p < 0.01), SCZ (p < 0.05) and HC (p < 0.01). For the X-coordinate, there was a significant trend (p < 0.1) for HC and MDD patients (Table S2). The correlation between the difference in Euclidean distance of XYZ electrode placement of investigator 2 minus investigator 1 and the difference between the number of significantly activated e-field voxels between investigator 2 minus investigator 1 showed a significant negative correlation for MDD with F3 (Pearson's r = −0.587, p = 0.002, 95% CI = −0.797, −0.25) and F4 electrode (Pearson's r = −0.607, p = 0.002, 95% CI = −0.808, −0.278) as well as SCZ with F3 (Pearson's r = −0.433, p = 0.031, 95% CI = −0.71, −0.05) and F4 (Pearson's r = −0.373, p = 0.066, 95% CI = −0.67–0.03). For the HC, a negative significant trend was observed at F3 (Pearson's r = −0.406, p = 0.067, 95% CI = −0.71–0.03) and F4 (Pearson's r = −0.386, p = 0.084, 95% CI = −0.70, 0.06) (Fig. S1).

### 3.5 Inter-individual difference

The standard deviation (SD) of the e-field value increased with raising the percentile threshold of the voxels. It indicates the inter-individual difference of the e-field intensity at higher-cap; the maximum e-field strength considerably varies inter-individually (Fig. 5A). Additionally, Fig. 5B shows three selected surface-based individual e-field models from each group, illustrating that there are individuals with relatively higher or lower e-fields. Even though there were significant cross-diagnostic differences, inter-individual differences within each group were noticeable.

## 4 Discussion

In a cross-diagnostic comparison of MDD and SCZ patients with HC, this study investigates the strength and distribution of individually modeled e-fields for bifrontal tDCS as applied in numerous clinical studies investigating therapeutic tDCS. To the best of our knowledge, this is the first study comparing tDCS-induced e-fields in patients with major psychiatric disorders and HC to test a basic assumption in the field, namely the translation of stimulation parameters from healthy subjects to clinical samples. Our main finding was that the average e-field strength considerably varied across subjects and was significantly lower in MDD and SCZ patients compared to HC. However, there was no significant difference in e-field intensity between both clinical samples. The difference between SCZ and HC was consistently found by both investigators for bilateral superior frontal gyrus and right middle frontal gyrus regions. Focusing on PFC ROIs, significant differences in e-field intensity between MDD and HC as well as SCZ and HC were consistently observed by both investigators for Sallet 8B and 9 regions, though the difference between MDD and SCZ did not reach statistical significance in any Sallet regions. In addition, there were marked differences in e-field intensities between investigators, though the number of two investigators does not allow to establish a valid estimate of inter-rater variability. On a descriptive level, we observed considerable inter-individual variability of e-field intensity within groups.

### 4.1 E-field intensity difference between clinical populations and healthy subjects

The present study showed that e-field intensity was lower in MDD and SCZ compared to HC with both whole-brain and PFC ROI-based analysis. E-field modeling was based on morphometric information from individual structural MRI scans. The changes in GM volumes and cortical thickness in clinical samples may contribute to the differences between clinical and non-clinical samples. There is comprehensive evidence of both GM atrophy in MDD (Chang et al., 2011, Du et al., 2014, Shah et al., 1998, Wise et al., 2017) and SCZ (Fornito et al., 2009, Théberge et al., 2007, Whitford et al., 2006) as well as reduced cortical thickness in MDD (Mackin et al., 2013, Rajkowska et al., 1999) and SCZ (Goldman et al., 2009, Narr et al., 2005, Rimol et al., 2010, Schultz et al., 2010, van Haren et al., 2011). However, the e-field differences between clinical populations and healthy controls are related to the structural MRI data and it does not inform about the overall translational validity from health to disease. A multitude of factors (e.g. neurochemical and molecular changes, functional network alterations, behavioral and cognitive differences) may impact the capacity for the translation even if e-field strengths are adjusted for clinical groups or individual patients.

In the light of our previous study that showed an association between the GM volume in the dorsal PFC and treatment outcome in the MDD (Bulubas et al., 2019), e-field modeling may play a future role in predicting NIBS outcome (Albizu et al., 2020, Suen et al., 2021). In SCZ, Mondino et al. (2021) recently reported that tDCS responders showed a higher e-field strength in the left transverse temporal gyrus at baseline compared to non-responders. Nevertheless, the interaction of e-fields with the individual anatomy is complex (Antonenko et al., 2021a). Though e-fields may represent a valid proxy for individually adjusted tDCS intensity, one has to keep in mind that dose–response relationships have not yet been established for tDCS and intensity is only one parameter of tDCS “dosage” which may not follow simplistic models.

### 4.2 Importance of precise electrode positioning

The current study used a python script that allowed two blinded investigators to calculate F3 and F4 positions based on nasion, inion, and mastoids coordinates. This approach imitated current clinical practice where operators use the international 10–20 EEG system for positioning tDCS electrodes over F3 and F4. Hence, it enabled us to investigate whether tDCS-induced e-fields differ between two investigators who determined the electrodes’ position and orientation independently. Though there was a high intraclass correlation between both investigators, there were also discrepancies in electrode positions and related e-field intensities. The spatial distribution of e-fields showed that investigator 1 tended to place the electrodes slightly differently than investigator 2, which is due to individual variation in implementation although the instruction was the same. Even such a small variation in positioning led to different statistical results derived from the discrepancies in the e-field distribution. In practice, this finding adds to the previous report by Opitz et al. (2018) who suggested limiting the electrode positioning error to be plus/minus 1 cm for achieving consistent results. The importance of precise electrode positioning must be emphasized here again because it can be easily forgotten as tES is a rather non-focal NIBS approach. Neuronavigation algorithms may represent valid approaches for precise electrode montages in tES applications (Jog et al. 2021).

### 4.3 Inter-individual variability of e-field

Another observation replicated from other studies (Seibt et al., 2015) is the considerable inter-individual differences in field strength and distribution which may be hidden under the group average. Even though there was a significant difference in group averages between clinical samples and HCs, it is noteworthy that there were patients who showed high-intensity e-fields comparable with HC, and there were HC subjects who showed low e-field strength at the level of other MDD and SCZ patients. Inter-individual differences in e-fields may be attributed to the variability of the treatment response, as e-fields intensity reflects the effect of the stimulation. Antonenko et al. (2021b) identified that head, skull, skin, and CSF volumes as anatomical variables explaining a major proportion of variability in general field strength and proposed to consider these parameters for empirical tDCS studies. As every brain has individual attributes which yield variability in e-fields, it is suggested to refer to the e-field information when deciding the stimulation parameters.

### 4.4 Individualization of tDCS

In conclusion, the question arises whether the fixed dosing (usually defined by x mA) for tES application should be replaced by individualized dosing regimes based on individual e-field modeling. Previous studies have shown that e-field intensity differs by more than 100% across subjects when electrodes are located at the conventional stimulation site of the primary motor cortex (Evans et al., 2020), and the inter-individual variability even increases further with focal montages (Mikkonen et al., 2020). Furthermore, the inter-individual difference in the e-field was associated with variability in the tES outcome (Kasten et al., 2019). When only group differences are considered, the inter-individual difference is masked by the group effect. For the future of tES, controlling for inter-individual and inter-rater variability will become a key to establishing more stable therapeutic effects.

### 4.5 Current limitation and future perspectives

The present study demonstrated the importance of the individualization of the tDCS protocol. Recent studies show that network-based approaches, such as e-field modeling or FC and connectome analyses with fMRI, contribute to the stratification or individualization of NIBS (Chen et al., 2018, Soleimani et al., 2021). E-field modeling is a relatively quick and simple but informative and meaningful method to define the stimulation dosage, electrode location, and montage depending on the individual brain structure. Additionally, since our two investigators showed different results, we suggest paying attention to the electrode localization variability occurring unconsciously, for example by using neuro-navigation.

Even though the stimulation strength and electrode locations are controlled, there are still other factors that are assumed to cause differences in the patient’s responsiveness. For example, brain state is a factor known to affect the ability to respond to tES but is difficult to control. Individual levels of fatigue, arousal, attention, anxiety, and excitement at the moment of the brain stimulation are potentially the confounding factors that modulate the outcome of the tDCS treatment (Krause and Cohen Kadosh, 2014). Additionally, circadian rhythm and hormonal levels could be also a source of variability (Krause and Cohen Kadosh, 2014). Since we have these almost uncontrollable sources of individual variability, we suggest at least controlling for e-field intensity for each individual.

The interpretation of our results should take into account the limitations that arise from the computational e-field modeling. First, the calculated e-field in this study is based on the individual anatomical features, meaning that it is only a proxy for the real e-field and stimulation. A kind of e-field validation can be obtained through in-vivo invasive electrophysiological recordings (Opitz et al., 2018, Wang et al., 2022). Second, in the present study, e-field modeling was based on the T1-weighted anatomical scans. However, it is recommended for future studies to also include T2-weighted images to improve the accuracy of CSF-skull segmentation. Lastly, to improve the quality of the e-field modeling, further research is needed to investigate the conductivity variance between individuals. With the current system, the conductivity is set at the same default value for all individuals. However, some previous studies showed that the calcification change related to aging causes a significant conductivity change in the skull for example (Hoekema et al., 2003, McCann et al., 2019).

Finally, our study has focused on one montage only, i.e. the standard F3-F4 montage used for therapeutic intervention in MDD (Bajbouj et al., 2018, Blumberger et al., 2012, Brunoni et al., 2013, Padberg et al., 2017). In the milestone ELECT-TDCS trial by Brunoni et al. (2017), a very similar montage has been applied to MDD, i.e. anode over left DLPFC and cathode over right DLPFC according to the Omni-Lateral Electrode (OLE) system (Seibt et al., 2015). In SCZ research, several studies have applied the F3 target for positioning the anode, however, the cathode position has been varied across studies (Palm et al., 2016, Valiengo et al., 2020). Recently, Antonenko et al. (2021a) applied SimNIBS modeling to six tDCS montages and introduced a measure of e-field focality determined by the area of the GM region with the field strengths higher than the 75th percentile, where higher values represent higher current spread, implying lower focality. In the second study, Antonenko et al. (2021b) compared four bipolar montages and four “focal” 4x1 montages and proposed the individual head circumference as a proxy for estimating individual differences in the tDCS induced e-field. Future studies should also investigate e-field parameters for different montages in comparison between clinical and non-clinical groups.

## 5 Conclusion

Our results revealed two important findings: 1) the mean strength of tDCS-induced e-fields based on the standard anode-F3/cathode-F4 bipolar montage is lower in MDD and SCZ compared to HC, but MDD and SCZ groups does not differ significantly either at the whole-brain level or on PFC ROI analysis. 2) Inter-individual and inter-rater differences are prominent and should not be ignored. The present study supports the hypothesis that dose–response relationships cannot be simply transferred from healthy cohorts and need to be specifically established for clinical groups, possibly using the MRI-based e-field strength as a proxy for individual dosing.

Further research is needed to develop predictors for therapeutic effects based on e-field models and to establish dose–response relationships for clinical applications.

## 6 Open Science Framework (OSF)

To support the Open Science approach and for transparency reasons, we have published our data at OSF: https://osf.io/74tz9/?view_only=eca19c741793403fa88379f4d30b979a.

## Funding

This work was supported by the German Center for Brain Stimulation (GCBS) research consortium (Work Package 5) [grant number 01EE1403E], funded by the Federal Ministry of Education and Research (BMBF).

### CRediT authorship contribution statement

**Yuki Mizutani-Tiebel:** Conceptualization, Methodology, Formal analysis, Investigation, Writing – original draft, Visualization. **Shun Takahashi:** Conceptualization, Methodology, Formal analysis, Investigation, Writing – review & editing. **Temmuz Karali:** Software. **Eva Mezger:** Conceptualization, Methodology. **Lucia Bulubas:** Conceptualization, Methodology. **Irina Papazova:** Investigation. **Esther Dechantsreiter:** Conceptualization, Methodology. **Sophia Stoecklein:** Investigation. **Boris Papazov:** Investigation. **Axel Thielscher:** Methodology, Supervision. **Frank Padberg:** Conceptualization, Methodology, Writing – review & editing, Supervision, Funding acquisition. **Daniel Keeser:** Conceptualization, Methodology, Formal analysis, Writing – review & editing, Visualization, Supervision, Project administration.

## Declaration of Competing Interest

The authors declare the following financial interests/personal relationships which may be considered as potential competing interests: The work of YM is part of a Ph.D. thesis of Munich medical research school, university hospital LMU. YM receives remuneration from neuroCare Group AG as a part-time office worker. AT is supported by the Innovation Fund Denmark (IFD) - grant agreement number 9068-00025B and was supported by the Lundbeck foundation (R244-2017-196 and R313-2019-622). FP is a member of the European Scientific Advisory Board of Brainsway Inc., Jerusalem, Israel, and the International Scientific Advisory Board of Sooma, Helsinki, Finland. He has received speaker's honoraria from Mag&More GmbH, the neuroCare Group, Munich, Germany, and Brainsway Inc. His lab has received support with equipment from neuroConn GmbH, Ilmenau, Germany, Mag&More GmbH and Brainsway Inc. Other authors reported no biomedical financial interests or potential conflicts of interest.
