# Supplementary material for: Differences in electric field strength between clinical and non-clinical populations induced by prefrontal tDCS: A cross-diagnostic, individual MRI-based modeling study
Source: Neuroimage Clin. 2022 Apr 16;34:103011. doi: 10.1016/j.nicl.2022.103011 (PMC9125784; doi:10.1016/j.nicl.2022.103011)
Supplement: Supplementary data 1 [file mmc1.docx]

**Supplementary Information**

Supplementary Table 1 and 2 show the statistical analysis of electrode locations F3 and F4, respectively. The following data are reported in the tables:

1) Mean and standard deviation (SD) of electrode locations for investigator 1 and 2. X, Y, Z coordinates are calculated on the individual native space. 2) Result of independent samples student t-test comparing the X, Y, Z coordinates of investigator 1 and 2. 3) Mean and SD of Euclidean distance between the center of the electrodes determined by investigator 1 and 2 for each group of subjects. The Euclidean distance between the xyz-coordinates was calculated using the following formula: $d=\sqrt{\left( X_{2}-X_{1} \right)^{2}+\left( Y_{2}-Y_{1} \right)^{2}+\left( Z_{2}-Z_{1} \right)^{2}}$

Supplementary Table S1: mean location of F3 electrode for the experimental groups

|  |  | Investigator 1 | Investigator 2 | Independent Sample T-test | | | | Euclidean Distance |
| --- | --- | --- | --- | --- | --- | --- | --- | --- |
|  |  | Mean (SD) | Mean (SD) | Statistic | df | p | Effect size | Mean (SD) |
| **HC** | X | -48.36 (±3.65) | -45.27 (±4.20) | -2.78 | 48 | <0.01 | -0.79 | 19.11 (±9.31) |
|  | Y | 52.32 (±10.10) | 38.16 (±9.90) | 5.008 | 48 | <0.001 | 1.42 |  |
|  | Z | 47.94 (±11.12) | 58.84 (±10.47) | -3.57 | 48 | <0.001 | -1.01 |  |
| **MDD** | X | -48.59 (±5.68) | -45.94 (±6.27) | -1.57 | 48 | 0.12 | -0.44 | 15.40 (±9.59) |
|  | Y | 49.29 (±10.33) | 41.26 (±11.36) | 2.62 | 48 | <0.05 | 0.74 |  |
|  | Z | 46.79 (±10.59) | 53.48 (±8.64) | -2.45 | 48 | <0.05 | -0.70 |  |
| **SCZ** | X | -51.37 (±5.66) | -48.06 (±4.81) | -2.18 | 46 | <0.05 | -0.63 | 13.89 (±6.39) |
|  | Y | 48.39 (±10.21) | 40.07 (±11.41) | 2.66 | 46 | <0.05 | 0.77 |  |
|  | Z | 47.45 (±8.96) | 54.88 (±9.26) | -2.83 | 46 | <0.01 | -0.82 |  |

*HC=healthy controls, MDD=major depressive disorder, SCZ=schizophrenia. SD= standard deviation. df= degree of freedom. XYZ are shown as group average for the experimental groups by both investigators. The Euclidean distance between the xyz-coordinates is calculated between the two Investigators.*

Supplementary Table S2: location of F4 electrode

|  |  | Investigator 1 | Investigator 2 | Independent Sample T-test | | | | Euclidean Distance |
| --- | --- | --- | --- | --- | --- | --- | --- | --- |
|  |  | Mean (SD) | Mean (SD) | Statistic | df | p | Effect size | Mean (SD) |
| **HC** | X | 49.67 (±4.37) | 47.44 (±4.85) | 1.71 | 48 | 0.10 | 0.48 | 19.50 (±9.18) |
|  | Y | 49.67 (±9.73) | 24.18 (±12.07) | 5.00 | 48 | <0.001 | 1.41 |  |
|  | Z | 51.13 (±11.54) | 60.67 (±10.97) | -3.00 | 48 | <0.01 | -0.85 |  |
| **MDD** | X | 51.56 (±6.74) | 48.51 (±5.75) | 1.72 | 48 | 0.09 | 0.49 | 16.30 (±10.30) |
|  | Y | 48.84 (±8.66) | 37.93 (±11.57) | 3.78 | 48 | <0.001 | 1.07 |  |
|  | Z | 47.78 (±9.33) | 55.39 (±7.95) | -3.10 | 48 | <0.01 | -0.88 |  |
| **SCZ** | X | 48.85 (±7.14) | 46.96 (±6.07) | 0.99 | 46 | 0.33 | 0.285 | 14.73 (±6.98) |
|  | Y | 48.12 (±8.30) | 37.32 (±10.12) | 4.04 | 46 | <0.001 | 1.17 |  |
|  | Z | 50.49 (±8.66) | 57.48 (±9.71) | -2.63 | 46 | <0.05 | -0.759 |  |

*HC=healthy controls, MDD=major depressive disorder, SCZ=schizophrenia. SD= standard deviation. df= degree of freedom. XYZ are shown as group average for the experimental groups by both investigators. The Euclidean distance between the xyz-coordinates is calculated between the two Investigators.*

To investigate the correlation between euclidean distance and simulated efield activity, the difference in XYZ coordinates with the difference between the two investigators and the significant activated e-field difference was directly examined. The greater the distance of the placed electrode between the two different investigators had been, the greater the e-field difference, see Figure S1.


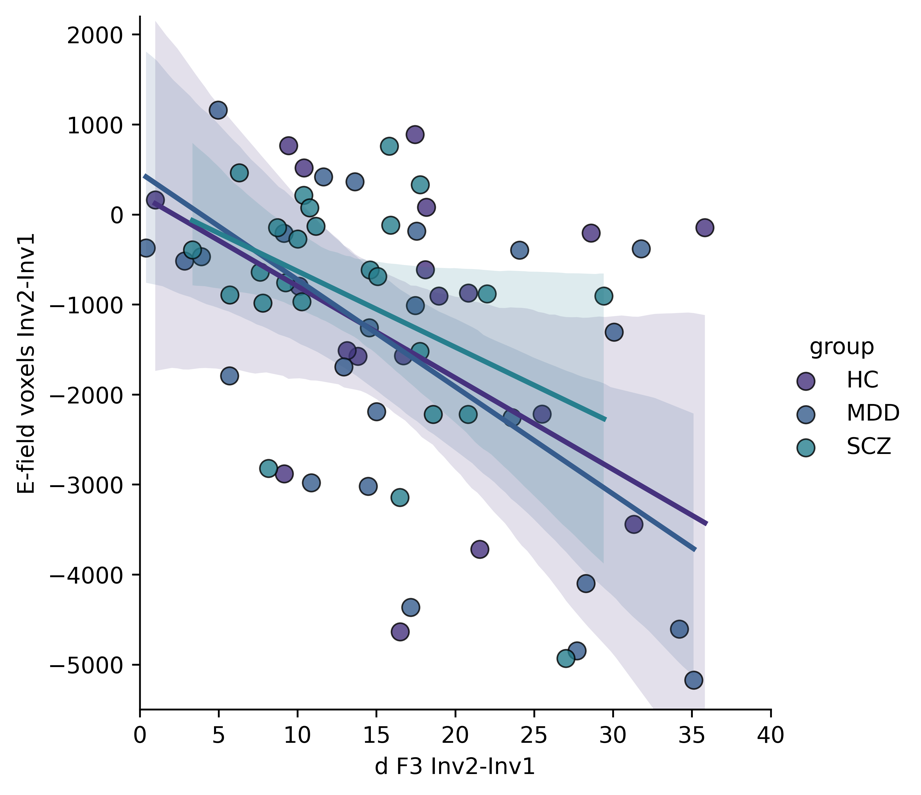


Figure S1:

*HC=healthy controls, MDD=major depressive disorder, SCZ=schizophrenia. d= Euclidean distance. y-axis E-field voxels difference between investigator 2 - investigator 1 in 2mm spatial resolution per voxel. x-axis=Euclidean distance d between Investigator 2 minus Investigator 1. Note: Across the three experimental groups HC, MDD, and SCZ, there was a significant negative correlation between e-field voxel activation and XYZ coordinate placement of the two investigators for electrode F3 (Pearson’s r= -0.512, p<0.001, 95% CI =, -0.67, -0.32) and F4 (Pearson’s r= -0.501, p<0.001, 95% CI =, -0.66, -0.30).*
